# A PCR-Based Molecular Detection of *Strongyloides stercoralis* in Human Stool Samples from Tabriz City, Iran

**DOI:** 10.3390/scipharm85020017

**Published:** 2017-03-27

**Authors:** Reza Ghasemikhah, Mohammad Amin Tabatabaiefar, Seyed Ali Shariatzadeh, Abbas Shahbazi, Teymour Hazratian

**Affiliations:** 1 Department of Parasitology& Mycology, School of Medicine, Arak University of Medical Sciences, Arak 3848176941, Iran; Dr.ghasemikhah@arakmu.ac.ir; 2 Department of Genetics and Molecular Biology, School of Medicine, Isfahan University of Medical Sciences, Isfahan 8174673461, Iran; Tabatabaiefar@gmail.com; 3 Departments of Parasitology and Mycology, School of Medicine, Tabriz University of Medical Sciences, Tabriz 3848176941, Iran; a.shariatzadeh@yahoo.com (S.A.S.); shahbazi42@yahoo.com (A.S.)

**Keywords:** *Strongyloides stercoralis*, molecular detection, PCR

## Abstract

*Strongyloides stercoralis* is a nematode causing serious infections in immunocompromised patients. In chronically infected patients, the low parasitic content as well as the resemblance of the larvae to several other species make diagnosis basedonmorphology difficult. In the present study, a PCR-based method targeting the internal transcribed sequence 2 (ITS2) of the rDNA region was examined for the molecular detection of *S. stercoralis* infection from the stool samples. A total of 1800 patients were included. Three fresh stool samples were collected per patient, and *S. stercoralis* isolates were identified by the morphological method. A subset of isolates was later used in the PCR-based method as positive controls. Additionally, negative and no-template controls were included. Data analysis was accomplished using an *x^2^* test. A*p*-value less than 0.05 was considered significant. In total, fivestool samples were found to be infected with *S. stercoralis* using the morphology method. PCR method detected *S. stercoralis* DNA target from all of the fiveDNA samples extracted from positive fecal samples. Conclusions: The PCR method used for amplifying a short fragment was successful for diagnosis of *S. stercoralis* in fecal samples and can be reliable for directly detecting the parasite bypassing morphological method.

## 1. Introduction

*Strongyloides stercoralis* is a human intestinal parasite distributed in temperate and tropical areas [1,2]. Current estimates suggest that between 30 and 100 million people are infected with *S. stercoralis* worldwide [3,4]. So far, various prevalence rates ranging from 4% to 50% have been reported from several regions around the world [5,6,7,8]. *S. stercoralis* has a unique life cycle, ensuring its survival in human hosts. Autoinfection occurs frequently, leading to chronic disease remaining for several years [3,9,10]. Hyper and disseminated infections may occur in immunocompromised patients which could result in death [11,12,13,14,15].

Definitive detection of strongyloidiasis is usually accomplished by virtue of the larval presence in human stool. However, in most cases, the intestinal worm load is very low leading to little recovery of the larvae in the stool. A number of methods have been used to detect larvae in fecal samples including the Baermann concentration method, formalin ethyl acetate concentration, agar plate culture (APC), and Harada–Mori culture. By conventional methods, the diagnosis rate would be low and several samples need to be examined to achieve appropriate sensitivity.

PCR-based assays have been employed to identify gastrointestinal worms [16]. Internal Transcribed Spacer (ITS) regions in the ribosomal DNA are repetitive sequences used for characterization of some pathogens including helminths in different studies [17,18].

Investigating the infection rate of *S. stercoralis* among the residents of Tabriz is of high importance. The city is in the northwest of Iran and has a special ecological context with much humidity desirable forthe distribution and transmission of parasites particularly intestinal ones [19].

In this study, a molecule-based approach was used to confirm morphologically diagnosed positive stool samples from Tabriz and to evaluate the utility of molecular diagnosis of *S. stercoralis.*

## 2. Materials and Methods

### 2.1. Subjects and Microscopic Diagnosis

The study was approved by the Review Board of Arak and Tabriz Universities of Medical Sciences (code number: 1388-88-02). From April 2012 to December 2012, a total of threestool samples per patient were collected from 1800 patients from hospital laboratories in Tabriz, Azerbaijan province, Northwest Iran. Informed consent was taken from participants before their inclusion in the study. All stool samples were directly examined under the microscope. Then, the samples were carefully examined using the formalin ether method.

Thelarvae obtained from the stool were washed extensively in distilled water and preserved in ethanol 70%. Larvae (L3) *S. stercoralis* were identified based on morphological characterizations using a light microscope and nematode taxonomic keys. Two independent parasitologists performed the morphological characterization of *S. stercoralis* to rule out similar nematodes such as *Rhabditis* spp.

### 2.2. DNA Extraction

About 3 g of each stool sample was preserved in 70% ethanol, treated by acetic acid and passed through the filter membrane and into the collection tube, towhich 3 mL of ether was added. Then, the tube was shaken gently and centrifuged at 1000× *g* for 2 min. The pellet was washed 2–3 times with distilled water and was ready for use in DNA extraction. DNA stool MiniKit of QIAamp^®^ (QIAGEN, Hilden, Germany) was applied to DNA extraction from larvae *S. stercoralis* in the stool. First, 1.4 mL of buffer ASL (a stool lysis buffer) was added to the sample and incubated in the 80 °C distilled water bath for 5 min. The procedure was continued following the manufacturer’s instructions to extract DNA from the stool. Elution Buffer (AE) (50 μL) was finally added to elute the extracted DNA. DNA quality was checked using a spectrometer (UNICO 2100, Dayton, NJ, USA) and 1.8% agarose gel stained with ethidium bromide.

### 2.3. Polymerase Chain Reaction

The ITS2 fragment of ribosomal DNA was PCR amplified using forward (SSF: 5′-ATCGTG TCGGTGGATCATTC-3′) and reveres (SSR: 5′-CTATTAGCGCCATTTGCATTC-3′) primer pair to obtain a specific 114 bp product [17]. PCR reactions were performed using the following reaction mixture: 2× red PCR mastermix (ROVALAB, Teltow, Germany), 25 pmol/μL of each primer, 1 μL of template, and distilled water up to the final volume of 25 μL under the following conditions: 1 cycle at 95 °C for 2 min (initial denaturation), 32 cycles of 94 °C for 30 s (denaturation), 58 °C for 30 s (annealing), and 72 °C for 45 s (extension), followed by a final extension at 72 °C for 3 min. For confirming the optimization process, DNA samples extracted from filariform larvae were used as positive controls and samples obtained to be negative by agar culture of stool and gastrointestinal parasites such as *Hymenolepis nana*, *Giardia lamblia*, and *Trichostrongylus colubriformis* and distilled water alone were used as negative controls.

### 2.4. Electrophoresis

PCR products were loaded on 1% agarose in Tris-EDTA-Boric acid (TBE) buffer (Bio life, Italina s.r.l, Milan, Italy). After an hour running at 80 V, the gels containing 0.5 μg/mL ethidium bromides (Roche, Berlin, Germany) were placed in a UV illuminator device to visualize the bands.

### 2.5. Statistical Analyses

Statistical analyses were carried out using the standard χ2 test, and where necessary, the Fisher exact test was used to estimate significance. *p*-Values < 0.05 were considered significant. All statistical analyses were performed using SPSS v.18.0 (IBM Co., Armonk, NY, USA).

## 3. Results

### 3.1. Microscopic Diagnosis

In total, 1800 stool samples were collected in this study and were examined using an optical microscope. Only twostool samples of larvae *S. stercoralis* were found under the microscope. However, the formalin ether results revealed fivepositive samples (Table 1).

### 3.2. DNA Extraction

All the extracted DNA samples passed the quality and quantity needed for PCR. They showed a single band on agarose electrophoresis. The 260/280 ratio was found to be >1.8.

### 3.3. PCR and Electrophoresis Results

**Figure 1:**
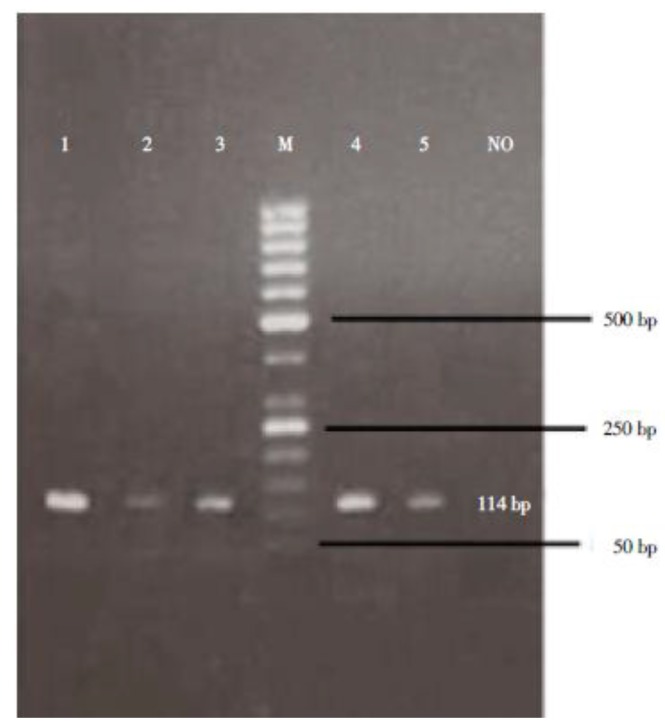
Agarose-gel electrophoresis of the single PCR products amplified by primers for a part of *S. stercoralis* rDNA. Lanes 1–3 and 4–5: PCR products of 5 positive stool samples; NO: no template sample; M: 50 bp DNA marker ladder.

Upon PCR amplification, all five samples successfully yielded the expected 114 bp product. No PCR amplification was detected in negative controls (Figure 1). Furthermore, 20 randomly selected negative stool samples were re-examined by molecular method, and all of them were confirmed to be negative.

## 4. Discussion

Investigating the infection rate of *S. stercoralis* among the residents of Tabriz city is of high priority due to the special ecological context of the city in Northwest Iran and to the potentially suitable conditions for distribution and transmission of parasites particularly intestinal parasites [19].

In the present study, a molecular approach was chosen to reliably confirm *S. stercoralis* samples and to help get insight into its prevalence in the studied samples. Based on the morphology approach, fivepositive (0.3%) cases were identified. The PCR assay was performed on the 5 morphologically diagnosed samples and 20 randomly selected negative samples. PCR confirmed all positive and chosen negative samples. The PCR assay was not utilized for all negative samples because the aim of the current study was to initially evaluate the ability of this molecular assay for diagnosis of *S. stercoralis.* The obtained prevalence (0.3%) for *S. stercoralis* was obtained to be lower compared to other studies from Iran. Mowlavi et al., 2007 [20] studied 379 people from tribal areas of Khuzestan province and reported the prevalence of *S. stercoralis* to be 0.6%. Rahimi-Esboei et al. [21] studied 4223 individuals from the central regions of Mazandaran province and found the prevalence of *S. stercoralis*to be 1.4%.

In the present study, PCR was applied as a highly sensitive technique to confirm and recheck the morphological result for diagnosis of strongyloidiasis. On the regular basis, diagnosis is made when the worm larvae are observed in fecal samples. However, the conventional technique is not always effective in detection of the parasite. It requires fresh stool samples and may be unable to detect very low amounts of the parasite. In addition, *S. stercoralis, Trichostrongyllus*, and *Rhabditis* are highly similar in term of morphological features and geographical distribution. This necessitates microscopy to be performed by askilledtechnician.

Efficient methods thatcan improve diagnosis chiefly in at-risk individuals are needed to help prevent potentially fatal infections [22,23]. PCR has been evaluated as a highly sensitive technique to detect protozoans and helminthic [15,24,25] infections in fecal samples.

This study is a successful validation of the ITS-2 PCR for detection of *S. stercoralis* obtained from human fecal samples. The results showed that this assay was able to detect all morphologically diagnosed samples. As a limitation of our study, PCR was not used for the majority of morphologically negative stool samples. The assay sensitivity and specificity should be determined ona broader scale in future studies. The technique has the potential to be used independently in future studies to more reliably detect this parasite.

## Figures and Tables

**Table 1:** No. of positive cases for *S. stercoralis* by direct exam and formalin ether methods.

*S. stercoralis*		Diagnosis Method
Negative (%)	Positive (%)	
1798 (99.9)	2 (0.1)	Direct exam
1795 (99.7)	5 (0.3)	Formalin ether
